# The Effectiveness and Safety of Chuna Manual Therapy Adjuvant to Western Medicine in Patients with Chronic Obstructive Pulmonary Disease: A Randomized, Single-Blind, Investigator-Initiated, Pilot Trial

**DOI:** 10.3390/healthcare12020152

**Published:** 2024-01-09

**Authors:** Jiwon Park, Minji Kwon, Beom-Joon Lee, Kwan-Il Kim, Hee-Jae Jung

**Affiliations:** 1 Department of Clinical Korean Medicine, College of Korean Medicine, Graduate School, Kyung Hee University, Seoul 02447, Republic of Korea; wldnjdjs219@khu.ac.kr (J.P.); franchisjun@naver.com (B.-J.L.); 2 Department of Clinical Pharmacology and Therapeutics, Kyung Hee University Hospital, Seoul 02447, Republic of Korea; 26041@khmc.or.kr; 3 Division of Allergy, Immune and Respiratory System, Department of Internal Medicine, College of Korean Medicine, Kyung Hee University, Kyung Hee University Medical Center, Seoul 02447, Republic of Korea

**Keywords:** chronic obstructive pulmonary disease, Chuna, effectiveness, safety, add-on

## Abstract

Recently, non-pharmacological treatments are gaining increasing importance for improving the quality of life in patients with chronic obstructive pulmonary disease (COPD). This pilot study aimed to evaluate the feasibility of conducting extensive research on Chuna manual therapy (CMT). This study investigated the effectiveness and safety of CMT adjuvant to Western medicine (WM) in patients with COPD. Forty patients with COPD were randomized into two groups in a 1:1 ratio: experimental (CMT plus WM) and control (WM only) groups. The CMT intervention was administered once a week for eight weeks. The primary outcome measured was the 6-min walk distance (6MWD). Secondary outcomes measured were: forced expiratory volume in 1 s (FEV1), forced vital capacity (FVC), assessments using the modified Medical Research Council (mMRC) scale and Visual Analog Scale (VAS) for dyspnea, the COPD Assessment Test (CAT), St. George’s Respiratory Questionnaire (SGRQ), and the EuroQoL five-dimensional questionnaire (EQ-5D). The mean differences in FEV1 (L) between Weeks 1 and 8 were statistically significant between the groups (*p* = 0.039). Additionally, the experimental group showed improved 6MWD, mMRC, VAS for dyspnea, CAT, SGRQ (total), and EQ-VAS scores than the control group. However, the differences between the two groups were not statistically significant. No adverse events were observed during this trial. CMT has the potential to alleviate symptoms, improve quality of life, and delay the decline in lung function in patients with COPD. The results of this pilot study could lead to large-scale clinical trials in the future.

## 1. Introduction

Chronic obstructive pulmonary disease (COPD) is characterized by chronic respiratory symptoms such as dyspnea, coughing, and phlegm production, caused by abnormalities in the airways or alveoli that induce persistent and progressive airflow obstruction [1]. The global incidence of COPD is 10.3% and is higher among people aged ≥60 years and smokers [2]. The aging global population and continued exposure to risk factors for COPD have resulted in an increased incidence of COPD, leading to significant economic and social burdens [3].

COPD treatment focuses on symptomatic relief, enhancing the quality of life, preventing exacerbations, and slowing down the progression of lung function decline [4]. Currently, inhalation therapy is the primary treatment for stable COPD, with priority given to the use of long-acting bronchodilators. Inhaled corticosteroids (ICS) may be used in conjunction if symptoms are not controlled [5]. Inhaled medications have been shown to effectively alleviate respiratory symptoms, enhance quality of life, and prevent exacerbations in patients with COPD [6,7,8]. However, the use of inhaled medications can be challenging and complex, and it may not be feasible for certain patients. Additionally, currently approved medications have limited mechanisms of action [9]. Pharmacotherapy alone cannot completely cure respiratory symptoms or prevent the decline in lung function. Furthermore, owing to the lifelong nature of COPD treatment, relying solely on pharmacotherapy is not economically sustainable and can lead to long-term side effects [5]. For example, prolonged use of inhaled corticosteroids has been linked to a higher risk of pneumonia, tuberculosis, oral candidiasis, and diabetes [10]. Therefore, the importance of non-pharmacological treatments such as education, physical activity, and pulmonary rehabilitation are being increasingly emphasized for improving the quality of life of patients with COPD [4].

Patients with COPD have hyperinflated lungs, which reduces the mobility of the thoracic spine and increases respiratory workload, leading to a compensatory increase in chest wall rigidity [11]. The associated reduced mobility of the chest wall leads to inefficient breathing, which subsequently results in reduced lung volume and dyspnea [12]. Manual therapy (MT) is a clinical procedure involving the direct stimulation of the spine, joints, and fascia using hands-on techniques. It increases joint mobility, reduces muscular hypertonicity and is typically associated with mild side effects [13]. Thus, MT can help alleviate chest wall rigidity, improve pulmonary function, and reduce dyspnea in patients with COPD [14]. Exercise tolerance is an important predictor of the quality of life and survival rates in patients with COPD. Their prognosis can be improved by mitigating exercise-induced dyspnea and enhancing physical capabilities [11].

Chuna manual therapy (CMT), a form of MT performed across Asia, incorporates the holistic concepts of traditional Korean medicine in its treatment approach [15]. CMT is used to treat various conditions, including impairment of respiratory and circulatory function, reduced mobility of the joints and muscles, and body misalignment. CMT can effectively treat not only musculoskeletal diseases but also tinnitus, gastric diseases, coughing, and rhinitis [16,17,18,19,20]. The CMT employed in this study is a method applied as a simple massage technique, commonly used in Korea. To date, no studies have examined the effectiveness and safety of CMT in patients with COPD. Therefore, prior to conducting a large-scale randomized controlled trial (RCT), a pilot study was conducted to ensure the successful implementation of larger RCT research. The objectives of this pilot study were to obtain preliminary data on the effectiveness and safety of CMT therapy for COPD, to perform sample size calculations for subsequent larger studies based on these findings, and to assess the feasibility of a full-scale study.

## 2. Materials and Methods

### 2.1. Study Protocol

The protocol of this study was registered at the Korean Clinical Trial Registry (http://cris.nih.go.kr (accessed on 8 April 2021); registration number: KCT0006119) and has already been published [21]. This study was approved by the Institutional Review Board of the Kyung Hee University Korean Medicine Hospital (KOMCIRB 2020-12-006-002). This clinical trial protocol complied with the Declaration of Helsinki and the GCP Guidelines. Informed consent was obtained from all the participants. This study was conducted in accordance with the Consolidated Standards of Reporting Trials (CONSORT) checklist (Table S1).

### 2.2. Study Design

**Figure 1:**
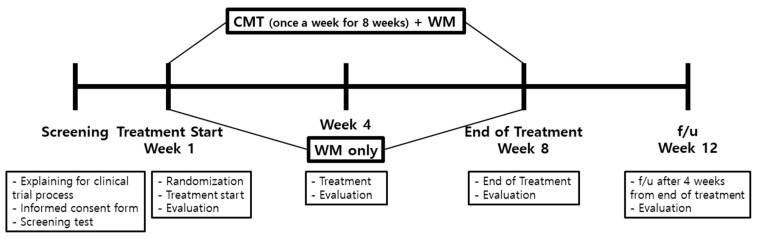
Research process of the study. CMT: Chuna **manual** therapy; WM: Western medicine; f/u: follow-up.

This prospective, randomized, single-blind, investigator-initiated pilot trial was conducted at the Kyung Hee University Korean Medicine Hospital. Patients receiving Western medicine therapy (WM) for COPD were randomized into two groups: the experimental group for a combination of CMT and WM, and the control group for WM only. The CMT intervention was administered once a week for 8 weeks, and assessments were performed at baseline and after 4, 8, and 12 weeks of intervention. The protocol has already been published [21] and a flow diagram of the study is shown in Figure 1.

### 2.3. Participants

#### 2.3.1. Inclusion Criteria

1. Patients aged ≥40 years and ≤80 years
2. Meeting the diagnostic criteria for COPD: Patients with a spirometry test result showing a forced expiratory volume in 1 s (FEV1)/forced vital capacity (FVC) ratio of <0.70 [4] and currently undergoing standard drug therapy
3. Patients who willingly provided informed consent to participate in the clinical trial

#### 2.3.2. Exclusion Criteria

1. Individuals with moderate-to-severe respiratory symptoms due to diseases other than COPD (cystic fibrosis, pneumonia, interstitial lung disease, and lung cancer)
2. Individuals with a history of alcohol or other substance abuse or dependency
3. Individuals diagnosed with a clinically significant disease or condition of the liver or heart, or cardiovascular, respiratory, endocrine, or central nervous system based on clinical testing, or having a history of malignant tumors or mental disorder. (However, patients were considered as eligible for participation if their conditions had not recurred for at least five years after surgery)
4. Individuals showing a change in drug use within the past three months prior to the present study
5. Patients who cannot walk without assistance
6. Patients who received exercise therapy, physiotherapy, or MT related to respiratory rehabilitation in the last month
7. Patients under oxygen therapy
8. Individuals contraindicated for Chuna MT
9. Pregnant or planning for pregnancy
10. Patients deemed unsuitable for participation in this study by the principal investigator

### 2.4. Randomization and Blinding

Participants were randomly assigned to either the experimental group or the control group in a 1:1 ratio. A statistician who was not directly involved in this study used the SAS Ver. 9.1.3, for Microsoft Windows (SAS Institute Inc., Cary, NC, USA) to assign random numbers to the participants and generate a random number table. The random number table generated using SAS and the allocation table for the experimental and control groups were maintained by a statistician until the end of the study, and the principal investigator did not access them.

The participants were assigned a screening number based on the order in which they signed the consent form. Participants who were ultimately selected for further evaluation after undergoing a screening test were assigned a registration number and grouped based on their subject registration number obtained from a random number table maintained by independent statisticians.

In this single-blind study, CMT was performed as an add-on therapy to standard drug therapy. The control group received the standard drug therapy only. Neither the participants nor the Korean medical doctors who performed the CMT were blinded to the group allocation. The principal investigator and researcher responsible for data collection were blinded to the group assignment.

### 2.5. Interventions

The CMT used in this study targeted the primary and accessory respiratory muscles. Steps 1–10 were performed uniformly for all the participants. Three Korean medical doctors, each with more than three years of experience, conducted the CMT sessions. Each session lasted 15 min. The instructions were as follows:

1. Cervical relaxation: The therapist assumed a position in which both the hands were wrapped around the patient’s neck while the patient was in the supine position. Using the second and third fingers, strong pressure was applied to the C1-7 spinous process and splenic muscles while rotating the neck.
2. Occipitocervical junction relaxation: The patient’s head was positioned outside the bed and firm pressure was applied on the occipitocervical junction using the second, third, and fourth fingers.
3. Trapezius muscle relaxation: Using the thumb and index finger, both trapezius muscles were grasped in a manner similar to when using forceps. Firm pressure was applied with the thumbs to promote muscle relaxation.
4. Clavicle relaxation: The upper and lower parts of the clavicle were pressed with the thumb with a rubbing motion.
5. Pectoralis major and latissimus dorsi muscle relaxation: With hands extended and the fingers stretched out, the therapist relaxed the patient’s pectoralis major muscle by applying force to the thumbs. Subsequently, the insertion point of the serratus anterior muscle was pressed vertically below the armpit using the thumb.
6. Rectus abdominal muscle relaxation (via the upper limbs): The therapist held both hands of the patient and extended the arms upward. Patients were instructed to fully extend their feet while exhaling (Set 1) and bring their toes toward the body while exhaling (Set 2), and the process was repeated.
7. Quadratus lumborum and intercostal muscle relaxation (via the upper limbs): The patient’s arms were stretched to the left while assuming the position from Step 6. Simultaneously, the direction of the patient’s feet was guided toward the left (Set 1). Subsequently, the patient’s arm was stretched to the right (Set 2). Simultaneously, the direction of the patient’s feet was guided toward the right (Set 2). The processes under Sets 1 and 2 were repeated.
8. Thoracic breathing relaxation (via pressure on the humeral head): The therapist placed their palms on both humeral heads and applied pressure while the patient inhaled. The pressure was released while the patient exhaled.
9. Breathing relaxation (via pressure on the pectoralis major muscle): The palms were placed on both the pectoralis major muscles, and pressure was applied to the muscles when the patient inhaled and released when the patient exhaled.
10. Abdominal trapezius and thoracolumbar paraspinal muscle relaxation: Pressure was continuously applied and released using both palms along the thoracolumbar paraspinal muscles, starting from the Dazhui point and progressing toward the waist. This step mainly focused on relaxing the abdominal trapezius and thoracolumbar paraspinal muscles.

### 2.6. Outcome Measures

The primary outcome metric of this study was the difference in the 6-min walk distance (6MWD) between the two groups after Week 8 compared with the baseline. The FVC and FEV1 were measured using spirometry, and the FEV1/FVC ratio was treated as the secondary outcome. To assess symptom severity and quality of life, we used the modified Medical Research Council (mMRC) scale [22] and Visual Analog Scale (VAS) for dyspnea, the COPD Assessment Test (CAT) [23], St. George’s Respiratory Questionnaire (SGRQ) [24], EuroQoL and the five-dimensional questionnaires (EQ-5D) and EQ-VAS [25] were used. The pulmonary function test (PFT) was performed at baseline and after 8 weeks of intervention, while the other assessments were performed at baseline and after 4, 8, and 12 weeks of intervention.

### 2.7. Sample Size Calculation

This was a prospective small-scale pilot clinical trial. We referred to commonly accepted practices, such as the rule of thumb proposed by Julious in 2005 [26] and Hertzog’s study [27]. To perform a sample size calculation for a larger trial, the minimum number of patients required to determine the initial data for the primary outcome measure, in order to perform a sample size calculation for a larger trial, was determined to be 16. Considering a 20% dropout rate, the total number of subjects for the trial was calculated to be 40.

### 2.8. Adverse Events

Adverse events (AEs) are defined as any undesired or unintended signs, symptoms, or diseases occurring during the course of a clinical trial. Throughout the study period, all the participants were continuously monitored for the occurrence of AEs. All AEs that occurred during the study were reported and documented according to the Common Terminology Criteria for Adverse Events (CTCAE).

### 2.9. Statistical Analysis

Continuous variables are presented as means with standard deviations, and categorical variables are represented as frequency numbers with percentages. The Wilcoxon rank-sum and chi-square tests were used to compare the treatment and control groups for continuous and categorical variables, respectively. Fisher’s exact test was performed when >25% of the cells had an expected frequency of <5 for continuous variables. Within each group, comparisons between the baseline (Week 1) and Week 8 were performed using the Wilcoxon signed-rank test. The mean difference was calculated as Week 8 minus Week 1, and the difference between the two groups was tested using the Wilcoxon rank-sum test. Statistical significance was set at *p*-value < 0.05. The effect size was measured using Cohen’s d to provide a measure of the magnitude of the treatment effect [28], with 0.2 indicating a small effect, 0.5 a medium effect, and 0.8 or greater indicating a large effect [29]. We included a measure of effect size because pilot studies such as this often have limited power to detect statistically significant differences, even when the findings are clinically relevant. Statistical analysis was conducted based on intention-to-treat analysis imputed by the method of the last observation carried forward (LOCF). All statistical analyses, including visualization using ggplot2 packages [30], were conducted using R (version 4.2.2) [31].

## 3. Results

### 3.1. Participants

**Figure 2:**
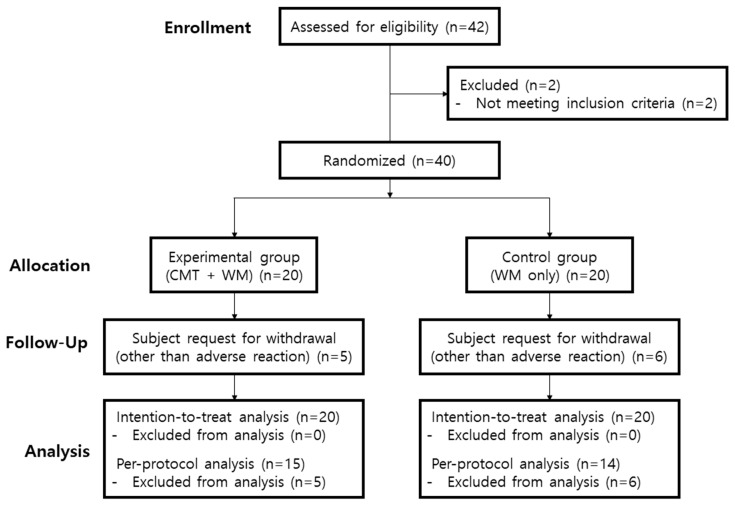
Flow chart of the study participants. CMT: Chuna manual therapy; WM: Western medicine.

In this study, participants were recruited from 17 May 2021 to 12 May 2022, and were followed up from 18 May 2021 to 25 July 2022. Based on the inclusion and exclusion criteria, 40 participants were enrolled in the clinical trial, and 20 were randomly assigned to the experimental group while the remaining 20 went to the control group. Of the 40 randomized patients, 29 (72.5%) completed the treatment. The completion rates of the experimental and control groups were 75% and 70%, respectively. (Figure 2). Table 1 shows the baseline characteristics of the 40 participants. There were no statistically significant differences between the two groups at baseline, except for sex and FVC (L) (*p* < 0.05). Among the nine discontinued participants, all but one dropped out after four weeks of initiating the trial.

### 3.2. Primary Outcome

**Figure 3:**
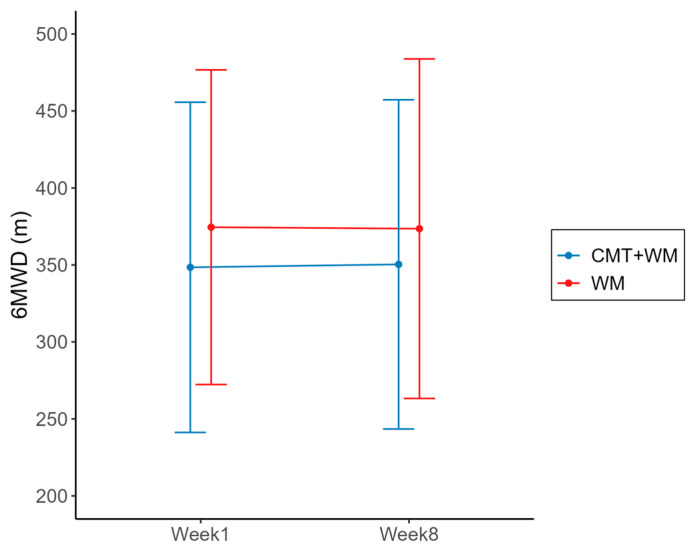
Results for 6MWD (m) from Week 1 to Week 8. 6MWD, 6-min walk distance; CMT: Chuna manual therapy; WM: Western medicine.

The primary outcome of this study, 6MWD, is presented in Table 2 and Figure 3. The mean differences in 6MWD from baseline (Week 1) to the primary endpoint (Week 8) were not statistically significant between the groups (*p* = 0.7548). The treatment effect was small (Cohen’s d = 0.0407). However, in contrast to the control group, in which the 6MWD decreased from Weeks 1 to 8, the 6MWD increased in the experimental group. For the full analysis set, the mean difference in the 6 MWD from Weeks 1 to 8 increased by 1.90 (−22.00, 23.80) in the experimental group and decreased by 0.95 ± 87.19 (−1.7, 39.86) in the control group.

### 3.3. Secondary Outcomes

**Figure 4:**
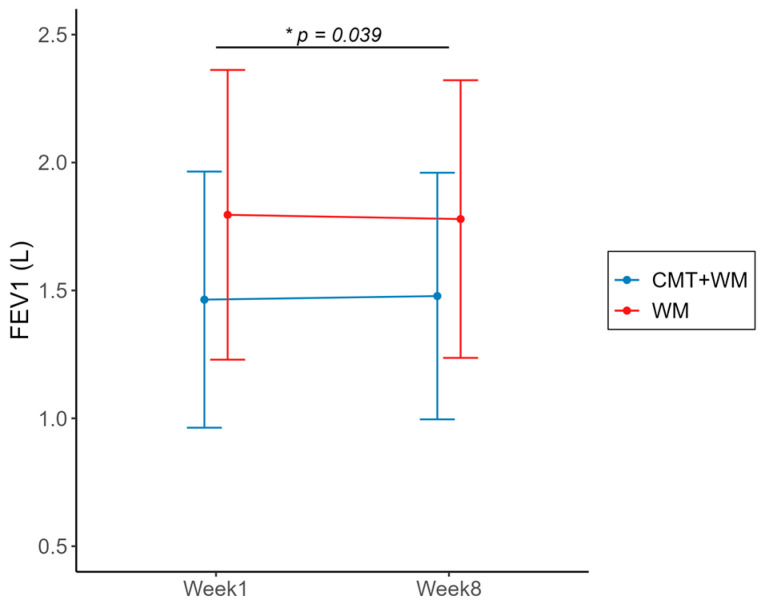
Results for FEV1 (L) from Week 1 to Week 8. FEV1, forced expiratory volume in one second; CMT: Chuna manual therapy; WM: Western medicine. Within each group, the mean difference was calculated as Week 8 minus Week 1, and the difference between the two groups was tested using the Wilcoxon rank-sum test. * *p*-value < 0.05.

**Figure 5:**
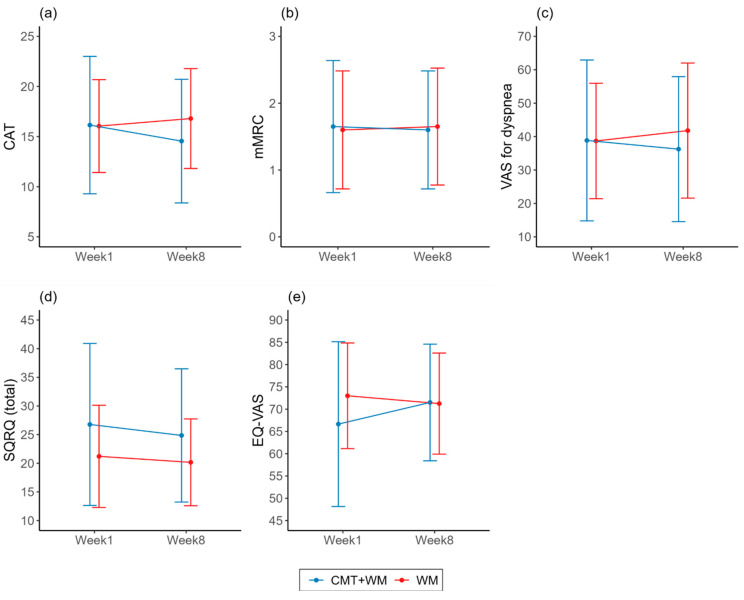
Results for secondary outcome variables from Week 1 to Week 8: (**a**) COPD Assessment Test (CAT), (**b**) modified Medical Research Council (mMRC), (**c**) Visual Analog Scale (VAS) for dyspnea, (**d**) St. George’s Respiratory Questionnaire (SGRQ) (total), (**e**) EuroQOL Visual Analog Scale (EQ-VAS). CMT: Chuna manual therapy; WM: Western medicine.

The secondary outcomes are presented in Table 2. Figure 4 and Figure 5 display some of these data. The mean difference in the FEV1 (L) from Week 1 to Week 8 increased by 0.01 (−0.02, 0.05) in the experimental group, and decreased by 0.02 ± 0.10 (−0.07, 0.03) in the control group. The mean differences in FEV1 (L) from Weeks 1 to 8 were statistically significant between the groups (*p* = 0.039) (Figure 4). The treatment effect was small (Cohen’s d = 0.34). The mean differences in CAT, mMRC, VAS for dyspnea, SGRQ (total), and EQ-VAS scores from Week 1 to Week 8 were not statistically significant between the groups but showed a certain trend. The mean difference in CAT scores from Weeks 1 to 8 decreased by 1.60 (−4.17, 0.97) in the experimental group, and increased by 0.75 (−0.78, 2.28) in the control group. The magnitude of the treatment effect was moderate (Cohen’s d = 0.5198). The mean difference in mMRC scores from Weeks 1 to 8 decreased by 0.05 (−0.23, 0.13) in the experimental group and increased by 0.05 (−0.23, 0.33) in the control group. The magnitude of the treatment effect is small (Cohen’s d = 0.1959). The mean difference in the VAS for dyspnea from Weeks 1 to 8 decreased by 2.6 (−14.53, 9.33) in the experimental group, and increased by 3.11 (−4.11, 10.31) in the control group. The treatment effect was small (Cohen’s d = 0.2707). The mean difference in the SGRQ (total) from Weeks 1 to 8 decreased by 1.91 (−4.26, 0.43) in the experimental group, and decreased by 1.05 (−3.81, 1.71) in the control group. The treatment effect was small (Cohen’s d = 0.1586). The mean difference in the EQ-VAS from Weeks 1 to 8 increased by 4.85 (−1.79, 11.49) in the experimental group, and decreased by 1.75 (−5.86, 2.36) in the control group (Figure 5). The magnitude of the treatment effect was moderate (Cohen’s d = 0.5595). The 6 MWD, CAT, mMRC, VAS for dyspnea, SGRQ (total), and EQ-VAS were obtained at Weeks 4 and 8 (after the last CMT session), and Week 12 (four weeks post-treatment). The changes in these values over time are presented in Figure S1.

### 3.4. Adverse Events

The vital signs and occurrence of AEs were assessed at each visit. All participants had normal vital signs and no AEs. C-reactive protein (CRP) levels were evaluated at Weeks 1 and 8. No abnormal C-reactive protein levels were observed in the CRP test results.

## 4. Discussion

To the best of our knowledge, this is the first pilot study to evaluate the efficacy and safety of concurrent CMT and WM in patients with COPD. Using a small sample size, we collected preliminary data on the effectiveness and safety of CMT in COPD. In this pilot study, significant differences were observed between the groups in several variables assessing efficacy, particularly FEV1 (L). Additionally, improvements in the 6 MWD, CAT, mMRC, VAS for dyspnea, SGRQ (total), and EQ-VAS scores were evident in the experimental group compared to the control group.

The 6MWD refers to the distance that a patient can briskly walk on a flat and firm surface within a duration of 6 min. It serves as an important measure of daily physical functioning and provides insights into a patient’s functional capacity [32]. Exercise tolerance is an important predictor of the quality of life and survival rate in patients with COPD. Although there was an increase in the walking distance in the CMT + WM group, the difference was minimal. Upon examining the research process, it was determined that a key variable affecting the study was the change in the individual measuring the 6 MWD during the course of the research. The subsequent measurement confirmed that, contrary to the commencement of the study, the walking distance was shortened by instructing the subjects to walk slowly. To address this issue in large-scale multicenter studies, standard operating procedures (SOPs) are being implemented to create accurate manuals for testers to eliminate bias.

FEV1 is an indicator of airflow limitation and is important for the diagnosis and managing of COPD [33]. The experimental group showed a statistically significant increase in FEV1 (L) compared to the control group, suggesting that CMT helps improve airflow limitation. Given the difficulties imposed by COVID-19, several participants were unable to undergo pulmonary function testing (PFT) at Week 8, resulting in the use of existing measurements. Considering the conservative nature of these results, it is reasonable to expect more promising results from large-scale clinical trials. Therefore, for a large-scale study, we intend to substitute FEV1 (L) as the primary outcome and determine the sample size using the FEV1 effect size.

The mMRC and VAS for dyspnea measure the severity of dyspnea in patients with COPD, and perceived dyspnea is highly correlated with quality of life [34]. The effect sizes for the mMRC and VAS were 0.1959 and 0.2707, respectively. The decreased mMRC and VAS scores in the experimental group indicate that CMT can relieve dyspnea and improve the quality of life. The quality of life in patients with COPD was assessed directly using the CAT, SGRQ, and EQ-VAS [35]. The observed decrease in the CAT and SGRQ (total) scores, along with the increase in the EQ-VAS score in the experimental group, suggests that CMT can improve the quality of life of patients with COPD. The effect sizes for the CAT, SGRQ, and EQ-VAS were 0.5198, 0.1586, and 0.5595, respectively. However, in the experimental group, none of the changes reached a minimal clinically important difference (MCID) [36,37,38], indicating the need for further confirmation through subsequent research.

The feasibility results of this study are as follows: This study was conducted as a CMT add-on trial, with the experimental group receiving visits once a week and the control group once every four weeks. The adherence rates in the experimental and control groups were 75% and 72.5%, respectively. In the control group, participants were randomly assigned and visited without receiving any intervention. Their dropout rate was not significant. The experimental group demonstrated high adherence, which led to the conclusion that the study design was appropriate. However, the patients who dropped out tended to do so after four weeks, emphasizing the importance of careful attention to the management of participants beyond the initial four-week period. COPD patients often experience respiratory and walking difficulties, prompting clinical interventions involving weekly CMT sessions. Reflecting on this, our research design incorporated weekly CMT interventions, totaling eight sessions over eight weeks. Once-weekly treatment sessions did not result in adherence issues. No issues arose, confirming the safety of CMT using simple myofascial relaxation techniques in elderly patients with COPD. Inclusion and exclusion criteria were not problematic. Considering that people cannot visit hospitals because of the pandemic, patient recruitment is expected to be feasible in the future. The sample size for the large-scale clinical trial was determined using FEV1(L). As this was an add-on trial without a placebo group, blinding between the experimental and control groups was not performed. Therefore, we opted for objective measurement tools for the primary variable, which is a crucial diagnostic indicator in patients with actual COPD, to ensure the reliability of the study results. Using an effect size of 0.3416, the sample size for this study was calculated. Finally, this pilot study provided a deeper understanding of the importance of quality management in clinical trials. As large-scale clinical trials are planned across multiple institutions, quality control is essential to ensure that procedures and methodologies are consistently implemented in a standardized manner across all participating institutions. To accomplish this objective, it is necessary to develop comprehensive guidelines that outline SOPs for clinical trials. Additionally, investigators and assessors participating in the trials should undergo standardized SOP training [39]. 

The limitations of this study include the recruitment of a small sample size from a single institution. This was inherent to the nature of the pilot study, and subsequent large-scale, multi-institutional research is planned to provide a more objective assessment of the efficacy and safety of CMT. Furthermore, as this study was an add-on trial of CMT, caution is warranted when interpreting the results because of the lack of blinding among the participants. Therefore, in the subsequent planned, large-scale study, we intend to establish a more objective primary outcome to reduce intergroup bias.

The CMT performed in this study targeted the primary and accessory respiratory muscles. Previous research suggests that CMT can enhance pulmonary function and alleviate dyspnea by improving chest wall compliance and thoracic movements [14]. Unlike respiratory rehabilitation, which focuses on active range of motion exercises, CMT emphasizes simple muscle relaxation. This approach, characterized by the avoidance of strenuous movements, is highly applicable to patients with severe COPD, thus enhancing its usability.

## 5. Conclusions

The present pilot study examined the effectiveness and safety of CMT in patients with COPD currently receiving WM and provided clinical evidence for a large-scale RCT to be conducted in the future. The results of this study serve as a basis for determining the optimal sample size for future large-scale studies. The data and results obtained from this pilot study are expected to inform future large-scale multicenter studies on the efficacy and safety of CMT in patients with COPD.

## Figures and Tables

**Table 1:** Baseline characteristics of participants.

Characteristic	CMT + WM (*n* = 20)	WM (*n* = 20)	*p*-Value
Age (years)	70.4 ± 7.11	70.9 ± 5.40	0.9675 *
Sex (male)	15 (75.0)	20 (100.0)	0.0471 *
Height (cm)	161.7 ± 7.1	165.5 ± 5.5	0.0619 *
Weight (kg)	62.8 ± 14.3	68.5 ± 14.3	0.2615 *
BMI (kg/m^2^)	23.8 ± 3.9	24.8 ± 4.35	0.6847 *
Smoking			
No	15 (75.0)	15 (75.0)	1 †
Yes	5 (25.0)	5 (25.0)	
Alcohol consumption			
No	20 (100.0)	19 (95.0)	1 ‡
Yes	0 (0.0)	1 (5.0)	
CRP			
normal (<0.5)	20 (100.00)	17 (85.00)	0.2308 ‡
NCS	0 (0.00)	3 (15.00)	
Primary outcome			
6MWD (m)	348.45 ± 107.23	374.50 ± 102.18	0.3866 *
Secondary outcomes			
CAT	16.15 ± 6.85	16.05 ± 4.63	0.839 *
mMRC	1.65 ± 0.99	1.60 ± 0.88	0.7899 *
VAS for dyspnea (mm)	38.85 ± 24.07	38.70 ± 17.27	0.8181 *
FEV1 (L)	1.46 ± 0.50	1.80 ± 0.57	0.1231 *
FEV1 (%)	55.1 ± 15.26	60.7 ± 16.35	0.2974 *
FVC (L)	2.70 ± 0.75	3.34 ± 0.75	0.0231 *
FVC (%)	71.40 ± 14.77	78.40 ± 14.59	0.213 *
FEV1/FVC (%)	54.50 ± 12.07	53.50 ± 10.72	0.7763 *
SGRQ (total)	26.77 ± 14.14	21.21 ± 8.92	0.2648 *
SGRQ (sysptoms component)	30.89 ± 12.77	30.32 ± 11.35	1 *
SGRQ (activity component)	45.69 ± 22.10	33.37 ± 13.48	0.0541 *
SGRQ (impacts component)	14.19 ± 13.05	11.01 ± 11.35	0.4043 *
EQ-5D (mobility)	2.45 ± 0.83	2.25 ± 0.55	0.4211 *
EQ-5D (self-care)	1.20 ± 0.52	1.05 ± 0.22	0.2985 *
EQ-5D (usual activities)	1.80 ± 0.89	1.60 ± 0.60	0.7144 *
EQ-5D (pain/discomfort)	1.60 ± 0.60	1.50 ± 0.51	0.6245 *
EQ-5D (anxiety/depression)	1.25 ± 0.72	1.10 ± 0.31	0.6206 *
EQ-VAS	66.65 ± 18.50	73.00 ± 11.85	0.2695 *

Data are presented as mean ± standard deviation or *n* (%). Statistical analysis was conducted based on intention-to-treat analysis imputed by the method of the last observation carried forward. CMT, Chuna manual therapy; WM, Western medicine; BMI, body mass index; CRP, C-reactive protein; NCS, not clinically significant; 6MWD, 6-min walk distance; CAT, COPD Assessment Test; mMRC, modified Medical Research Council; VAS, Visual Analog Scale; FEV1, forced expiratory volume in one second; FVC, forced vital capacity; SGRQ, St. George’s Respiratory Questionnaire; EQ-5D, EuroQOL five-dimensional questionnaire. *: *p*-value were derived from Wilcoxon rank-sum test; †: *p*-value were derived from Chi-squared test; ‡: *p*-value were derived from Fisher’s exact test.

**Table 2:** Mean differences of the experimental (WM plus CMT) and control (WM only) groups for primary and secondary outcome variables.

	CMT + WM (*n* = 20)			WM (*n* = 20)			*p*-Value †	Effect Size
	Week 1	Week 8	Mean Difference	Week 1	Week 8	Mean Difference		
6MWD (m)	348.45 ± 107.23	350.35 ± 106.90	1.90 (−20.00, 23.80)	374.50 ± 102.18	373.55 ± 110.25	−0.95 (−1.76, 39.86)	0.7548	0.0407
FEV1 (L)	1.46 ± 0.50	1.48 ± 0.48	0.01 (−0.02, 0.05)	1.80 ± 0.57	1.78 ± 0.54	−0.02 (−0.07, 0.03)	0.039 *	0.3416
FEV1 (%)	55.1 ± 15.26	55.7 ± 14.77	0.60 (−0.83, 2.03	60.7 ± 16.35	60.4 ± 16.22	−0.30 (−1.74, 1.14)	0.1234	0.2936
FVC (L)	2.70 ± 0.75	2.70 ± 0.69	0.00 (−0.06, 0.06)	3.34 ± 0.75	3.34 ± 0.71	0.00 (−0.06, 0.06)	0.231	0.012
FVC (%)	71.40 ± 14.77	71.55 ± 13.75	0.15 (−1.28, 1.58)	78.40 ± 14.59	78.60 ± 14.39	0.20 (−1.32, 1.72)	0.3168	0.0159
FEV1/FVC (%)	54.50 ± 12.07	54.85 ± 12.56	0.35 (−0.35, 1.05)	53.50 ± 10.72	53.10 ± 10.96	−0.40 (−1.21, 0.41)	0.5257	0.4638
CAT	16.15 ± 6.85	14.55 ± 6.17	−1.60 (−4.17, 0.97)	16.05 ± 4.63	16.80 ± 4.98	0.75 (−0.78, 2.28)	0.6289	0.5198
mMRC	1.65 ± 0.99	1.60 ± 0.88	0.05 (−0.23, 0.13)	1.60 ± 0.88	1.65 ± 0.88	0.05 (−0.23, 0.33)	0.7129	0.1959
VAS for dyspnea (mm)	38.85 ± 24.07	36.25 ± 21.69	2.60 (−14.53, 9.33)	38.70 ± 17.27	41.80 ± 20.21	3.10 (−4.11, 10.31)	0.524	
SGRQ (total)	26.77 ± 14.14	24.86 ± 11.63	−1.91 (−4.26, 0.43)	21.21 ± 8.92	20.16 ± 7.57	−1.05 (−3.81, 1.71)	0.0603	0.1586
SGRQ (symptoms component)	30.89 ± 12.77	31.14 ± 11.74	0.24 (−2.08, 2.57)	30.32 ± 11.35	30.30 ± 8.43	−0.02 (−2.92, 2.89)	1	0.0464
SGRQ (activity component)	45.69 ± 22.10	41.97 ± 18.56	−3.72 (−8.02, 0.59)	33.37 ± 13.48	34.03 ± 12.94	0.67 (−1.14, 2.47)	0.0583	0.6207
SGRQ (impacts component)	14.19 ± 13.05	12.64 ± 9.96	−1.55 (−4.27, 1.18	11.01 ± 11.35	8.61 ± 7.06	−2.40 (−7.37, 2.57)	0.5237	0.0996
EQ-5D (mobility)	2.45 ± 0.83	2.20 ± 0.52	−0.25 (−0.62, 0.12)	2.25 ± 0.55	2.15 ± 0.37	−0.10 (−0.31, 0.11)	0.654	0.2345
EQ-5D (self-care)	1.20 ± 0.52	1.10 ± 0.31	−0.10 (−0.31, 0.11)	1.05 ± 0.22	1.15 ± 0.37	0.10 (−0.04, 0.24)	0.1117	0.521
EQ-5D (usual activities)	1.80 ± 0.89	1.60 ± 0.60	−0.20 (−0.56, 0.16)	1.60 ± 0.60	1.65 ± 0.59	0.05 (−0.27, 0.37)	0.3301	0.3433
EQ-5D (pain/discomfort)	1.60 ± 0.60	1.60 ± 0.50	0.00 (−0.26, 0.26)	1.50 ± 0.51	1.45 ± 0.51	−0.05 (−0.29, 0.19)	0.7824	0.0931
EQ-5D (anxiety/depression)	1.25 ± 0.72	1.10 ± 0.31	−0.15 (−0.50, 0.20)	1.10 ± 0.31	1.05 ± 0.22	−0.05 (−0.15, 0.05)	0.9793	0.1818
EQ-VAS	66.65 ± 18.50	71.50 ± 13.09	4.85 (−1.79, 11.49)	73.00 ± 11.85	71.25 ± 11.34	−1.75 (−5.86, 2.36)	0.1254	0.5595

Data are presented as mean ± standard deviation. The mean difference was calculated as Week 8–Week 1. Statistical analysis was conducted based on intention-to-treat analysis imputed by the method of the last observation carried forward. CMT, Chuna manual therapy; WM, Western medicine; 6MWD, 6-min walk distance; CAT, COPD Assessment Test; mMRC, modified Medical Research Council; VAS, Visual Analog Scale; FEV1, forced expiratory volume in one second; FVC, forced vital capacity; SGRQ, St. George’s Respiratory Questionnaire; EQ-5D, EuroQOL five-dimensional questionnaire. †: *p*-value were derived from Wilcoxon rank-sum test; *: *p*-value < 0.05.

## Data Availability

The data that support the findings of this study are available from the corresponding author upon reasonable request.

## Supplementary Materials

The following supporting information can be downloaded from: https://www.mdpi.com/article/10.3390/healthcare12020152/s1, Table S1: CONSORT checklist; Figure S1: Results for primary and secondary outcome variables.
