# Supplementary material for: The Effectiveness and Safety of Chuna Manual Therapy Adjuvant to Western Medicine in Patients with Chronic Obstructive Pulmonary Disease: A Randomized, Single-Blind, Investigator-Initiated, Pilot Trial
Source: Healthcare (Basel). 2024 Jan 9;12(2):152. doi: 10.3390/healthcare12020152 (PMC10815833; doi:10.3390/healthcare12020152)
Supplement: Supplementary file 1 [file healthcare-12-00152-s001.zip › healthcare-2755424-supplementary.pdf]

Table S1. CONSORT checklist.

		Reporting Item	Page Number
**Title and Abstract**			
Title	#1a	Identification as a randomized trial in the title.	p.1
Abstract	#1b	Structured summary of trial design, methods, results, and conclusions	p.1
**Introduction**			
Background and objectives	#2a	Scientific background and explanation of rationale	p.1-2
Background and objectives	#2b	Specific objectives or hypothesis	p.2
**Methods**			
Trial design	#3a	Description of trial design (such as parallel, factorial) including allocation ratio.	p.2-4
Trial design	#3b	Important changes to methods after trial commencement (such as eligibility criteria), with reasons	N/A
Participants	#4a	Eligibility criteria for participants	p.3
Participants	#4b	Settings and locations where the data were collected	p.3
Interventions	#5	The experimental and control interventions for each group with sufficient details to allow replication, including how and when they were actually administered	p.4-5
Outcomes	#6a	Completely defined prespecified primary and secondary outcome measures, including how and when they were assessed	p.5
Outcomes	#6b	Any changes to trial outcomes after the trial commenced, with reasons	N/A
Sample size	#7a	How sample size was determined.	p.5
Sample size	#7b	When applicable, explanation of any interim analyses and stopping guidelines	N/A
Randomization - Sequence generation	#8a	Method used to generate the random allocation sequence.	p.4
Randomization - Sequence generation	#8b	Type of randomization; details of any restriction (such as blocking and block size)	N/A

Randomization - Allocation concealment mechanism	#9	Mechanism used to implement the random allocation sequence (such as sequentially numbered containers), describing any steps taken to conceal the sequence until interventions were assigned	p.4
Randomization - Implementation	#10	Who generated the allocation sequence, who enrolled participants, and who assigned participants to interventions	p.4
Blinding	#11a	If done, who was blinded after assignment to interventions (for example, participants, care providers, those assessing outcomes) and how.	p.4
Blinding	#11b	If relevant, description of the similarity of interventions	N/A
Statistical methods	#12a	Statistical methods used to compare groups for primary and secondary outcomes	p.5-
Statistical methods	#12b	Methods for additional analyses, such as subgroup analyses and adjusted analyses	N/A
**Results**			
Participant flow diagram (strongly recommended)	#13a	For each group, the numbers of participants who were randomly assigned, received intended treatment, and were analysed for the primary outcome	p.6 (Fig. 2)
Participant flow	#13b	For each group, losses and exclusions after randomization, together with reason	p.6
Recruitment	#14a	Dates defining the periods of recruitment and follow-up	p.6
Recruitment	#14b	Why the trial ended or was stopped	N/A
Baseline data	#15	A table showing baseline demographic and clinical characteristics for each group	p.6-7 (Table 1)
Numbers analysed	#16	For each group, number of participants (denominator) included in each analysis and whether the analysis was by original assigned groups	p.6-8 (Table 1, 2)
Outcomes and estimation	#17a	For each primary and secondary outcome, results for each group, and the estimated effect size and its precision (such as 95% confidence interval)	p.7-8 (Table 2)
Outcomes and estimation	#17b	For binary outcomes, presentation of both absolute and relative effect sizes is recommended	N/A
Ancillary analyses	#18	Results of any other analyses performed, including subgroup analyses and adjusted analyses, distinguishing pre-specified from exploratory	N/A

Harms	#19	All important harms or unintended effects in each group (For specific guidance see CONSORT for harms)	p.11
**Discussion**			
Limitations	#20	Trial limitations, addressing sources of potential bias, imprecision, and, if relevant, multiplicity of analyses	p.12
Generalisability	#21	Generalisability (external validity, applicability) of the trial findings	p.11-12
Interpretation	#22	Interpretation consistent with results, balancing benefits and harms, and considering other relevant evidence	p.11-12
**Other information**			
Registration	#23	Registration number and name of trial registry	p.2
Protocol	#24	Where the full trial protocol can be accessed, if available	p.2
Funding	#25	Sources of funding and other support (such as supply of drugs), role of funders	p.13

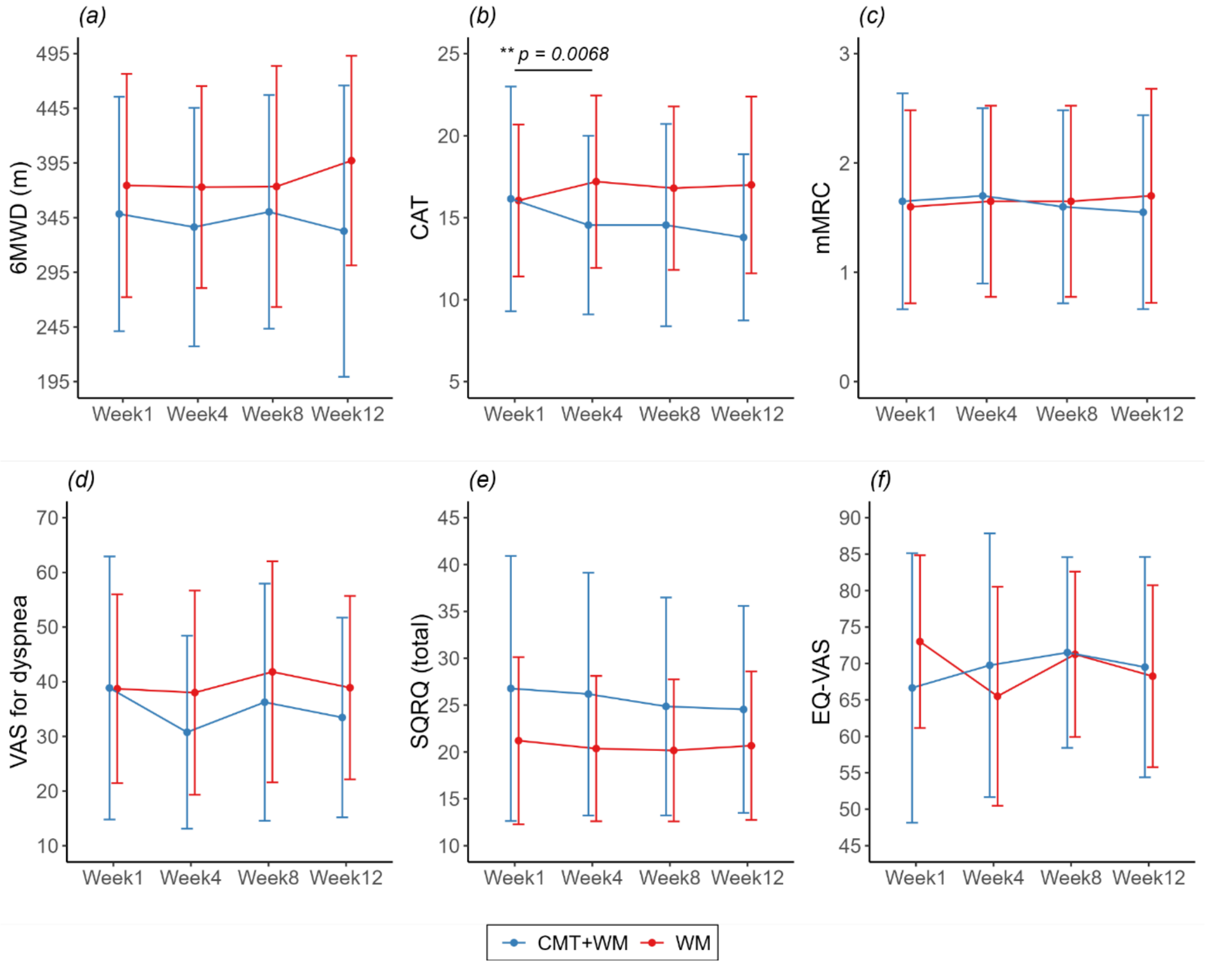

Figure S1. Results for primary and secondary outcome variables: (a) 6-minute walk distance (6MWD) (m), (b) COPD assessment test (CAT), (c) modified Medical Research Council (mMRC) scale, (d) visual analog scale (VAS) for dyspnea, (e) St. George's Respiratory Questionnaire (SGRQ) (total), (f) EuroQOL visual analog scale (EQ-VAS). CMT: Chuna manual therapy; WM: Western medicine. Within each group, the mean difference was calculated as week 4, 8, or 12 minus week 1, and the difference between the two groups was tested using the Wilcoxon rank-sum test. ** *p*-value < 0.01.
